# Contribution of the ethics committee of the French Intensive Care Society to describing a scenario for implementing organ donation after Maastricht type III cardiocirculatory death in France

**DOI:** 10.1186/2110-5820-2-23

**Published:** 2012-07-02

**Authors:** Jean-Pierre Graftieaux, Pierre-Edouard Bollaert, Lise Haddad, Nancy Kentish-Barnes, Gérard Nitenberg, René Robert, Daniel Villers, Didier Dreyfuss

**Affiliations:** 1 Département D’anesthésie-Réanimation, CHU de Reims, Hôpital Robert Debré, Avenue du Général Koenig, 51100, Reims, France; 2 Service de Réanimation Médicale; CHU de Nancy, Hôpital Central, 29 Av du Maréchal de Lattre de Tassigny, 54035, Nancy Cedex, France; 3 Consultation Douleur, CHU Saint-Louis, avenue Claude Vellefaux, 75010, Paris, France; 4 Service de Réanimation Médicale; CHU Saint-Louis, avenue Claude Vellefaux, 75010, Paris, France; 5 Service de Réanimation Médico-Chirurgicale, Institut Gustave Roussy, 39 rue Camille Desmoulins, 94805, Villejuif Cedex, France; 6 Service de Réanimation Médiclae, CHU de Poitiers, Hôpital Jean Bernard, 2 rue de la Milétrie, 86021, Poitiers Cedex, France; 7 Service de Réanimation Médicale, CHU de Nantes, Hôtel Dieu, Place Alexis Ricordeau, 44093, Nantes, Cedex 1, France; 8 Service de Réanimation Médico-Chirurgicale, CHU Louis Mourier, 92700, Colombes, France

**Keywords:** Organ donation, Treatment withdrawal, Cardiocirculatory arrest

## Abstract

French law allows organ donation after death due to cardiocirculatory arrest. In the Maastricht classification, type III non-heart-beating donors are those who experience cardiocirculatory arrest after the withdrawal of life-sustaining treatments. French authorities in charge of regulating organ donation (Agence de la Biomédecine, ABM) are considering organ collection from Maastricht type III donors. We describe a scenario for Maastricht type III organ donation that fully complies with the ethical norms governing care to dying patients. That organ donation may occur after death should have no impact on the care given to the patient and family. The dead-donor rule must be followed scrupulously: the organ retrieval procedure must neither cause nor hasten death. The decision to withdraw life-sustaining treatments, withdrawal modalities, and care provided to the patient and family must adhere strictly to the requirements set forth in patient-rights legislation (the 2005 Léonetti law in France) and should not be influenced in any way by the possibility of organ donation. A major ethical issue regarding the family is how best to transition from discussing treatment-withdrawal decisions to discussing possible organ retrieval for donation should the patient die rapidly after treatment withdrawal. Close cooperation between the healthcare team and the organ retrieval team is crucial to minimize the distress of family members during this transition. Modalities for implementing Maastricht type III organ donation are discussed here, including the best location for withdrawing life-sustaining treatments (operating room or intensive care unit).

## Review

### Principles

All procedures performed in the intensive care unit (ICU), regardless of the final outcome, are performed on and for a human being who has inherent and inalienable rights, regardless of his or her medical condition [1]. Compliance with the ethical requirements ensuring that these rights are respected underpins and legitimizes the technical healthcare procedures. Consequently, no technical requirements, however legitimate, can override the ethical requirements, failing which the patient would be a mere object to be used for a strictly technical activity devoid of humanity. These ethical requirements govern the activity of intensivists by framing their goal and mission, which consist in saving lives whenever possible and, otherwise, in providing continuous care until death occurs, under the best possible conditions. The goal and mission of organ retrieval teams are no less legitimate but differ from those of intensivists. Organ retrieval teams seek either to save the life of a person who will possibly, probably, or certainly die without organ transplantation or to improve the quality of life of a person who has a chronic disorder. The technical procedures needed to collect organs are inseparable from the underlying ethical requirements, including those dealing with the body of the donor (i.e., the obligation to perform reconstructive procedures that restore the human appearance of the body).

### What is a Maastricht type III donor?

In Maastricht type III donation [2] (also known as controlled donation after circulatory determination of death [DCDD]), cardiocirculatory arrest is awaited, as it results from the withdrawal of life-sustaining treatments. In this situation, cardiocirculatory arrest is not induced but is expected to occur, in a patient for whom no effective treatment options are available and as a result of a treatment-withdrawal decision made in agreement with advance directives (if available), the family, and the healthcare team. The occurrence of cardiocirculatory arrest shortly after treatment withdrawal may allow the collection of organs for transplantation (only the liver, kidneys [and cornea] are considered for collection in Maastricht type III donation by French regulatory authorities).

### Providing continuous end-of-life care takes precedence over organ retrieval

The withdrawal of treatments that have become useless, disproportionate, and unreasonable and whose only effect is to maintain life artificially should be performed in accordance with French law (the Léonetti law [3]): treatment withdrawal is not performed with the goal of allowing Maastricht type III donation. Treatment withdrawal aims to allow death to occur, that is, to avoid prolongation of the dying process by interventions that are useless, costly, and possibly degrading. The treatment-withdrawal decision can only be fully legitimate when placed in the clinical context, which is unique to each patient. As long as the patient is alive, he or she cannot be viewed as a potential reservoir of organs or other materials that could be put to use, failing which the patient would be robbed of his or her death and considered, not as a finality, but as a means put prematurely to use by others.

Giving priority to the desire to save lives via organ retrieval by instrumentalizing a dying patient at the expense of providing care and ensuring dignity throughout the dying process, as set forth in the Léonetti law, is ethically unacceptable. The issue of the precedence of one reason for acting (to save lives) over another (to provide continuous care to patients) raises the issue of the status of the individual. Lives in this case are perceived as able to replace one another, instead of biographic experiences that are unique to each individual. This position that views the human being from a utilitarian perspective, perhaps even as an instrument, converts the broad scope of life, which encompasses the dying process, to narrowly organic considerations. It may seem to be justified by the current severe shortage of organs, which causes considerable harm to patients who experience increasing ill health or who die because no transplant is available, as well as to the organ retrieval team, for whom the well-being and survival of transplant-list patients are crucial objectives. The argument that organ retrieval is justified because transplantable organs are in short supply suggests that these organs, and the lives they save, may have value independently from the person who donated the organs, as if one *had* a body instead of *being* a body.

For the ICU team, reasoning in terms of “potential organ donation” and, to an even greater extent, of “necessary preparations” before the end of the sequence of patient care required by treatment withdrawal contradicts ethical, psychological, and human principles in a way that is untenable. This contradiction must not be overlooked. Such a change in the paradigm of care during a sequence defined by its goal, unity, and cohesion would generate severe or even unbearable tensions among the healthcare team (physicians and other healthcare professionals), as well as mistrust and suspicion responsible for overwhelming anxiety among the family members and friends of the patient. This relinquishing of the principles of beneficence and nonmaleficence [4] to support a reckless view of the sole distributive justice principle would harm the dying patient and the family and also might adversely affect the alleged goal, namely, organ retrieval. Even the tiniest deviation from ethical principles during organ retrieval after cardiac arrest (e.g., hastening of ineludible cardiac arrest with the goal of ensuring optimal organ retrieval) would—and rightly so—attract the attention of the media and cause legitimate outrage among the general public. Any situation that is handled as justifying instrumentalization of the patient (beyond the unique features of the individual case generating the emotional reaction) would probably be counterproductive, not only for non-heart-beating donation but also for donation after brain death. No one would benefit from violating the bond of trust that underlies the organ retrieval and transplantation process.

### Moral validity of the actions taken by the intensive care unit team

The following points are crucial for the ICU team.

The team should act as if Maastricht type III donation did not exist. This attitude is a fabrication but constitutes a valuable firewall against ethical violations. The result is scrupulous adherence by the team to the separation between two different temporalities, namely, the dying process as it is allowed to unfold and the procedures performed to enable postmortem organ retrieval.

The team should be acutely aware of the difference between the death of the patient, in the biological and human meanings of the term, which is irreversible (as reflected by the entry on the death certificate indicating that death is permanent), and the dying process, during which the organs and body components stop functioning over a variable period and which continues to contribute to the meaning that will be given retrospectively to the patient’s life. The gap between these two concepts is the space in which collection of viable organs from the body of a dead person can occur.

### Which patients with withdrawal decisions may be candidates for organ donation after their death?

Organ donation may be considered if the following criteria are met:

The treatment-withdrawal decision was made independently from any considerations about the potential for subsequent organ donation, and

Death occurred immediately after treatment withdrawal.

Ideally, these patients should be defined by consensus among all the learned societies involved (SRLF, SFAR, and SFMU in France). Until such a consensus is achieved, the SRLF suggests considering potential organ donation after death in patients with head injuries, stroke, or anoxic brain injury with coma whose death seems unavoidable in the short term but who nevertheless do not progress to brain death.

For the moment, other patients with treatment-withdrawal decisions and the small number of patients who ask for treatment withdrawal are not considered to be potential donors. Indeed, in such situations the approach would have to be highly specific; in particular, any opinions expressed spontaneously by the patients would have to be taken into account. Consequently, the SRLF has decided not to take position on these situations for the time being.

### Importance of publicity and the national debate

At present, donors usually envision organ retrieval after a fatal accident. In this scenario, an unexpected event occurs, putting an end to a life during which the individual may have made decisions, such as carrying an organ donor card. Efforts are needed to raise awareness in the general public about the possibility of organ retrieval after death due to a devastating disease for which there is no reasonable hope for improvement but which may last for some time.

An information campaign is needed before the implementation of Maastricht type III donation to ensure that appropriate information is available to all those involved: the scenario must be accepted by the donors, the healthcare teams, and the recipients. A survey would probably be useful to identify any possible ethical stumbling blocks before implementing Maastricht type III donation.

The debate about explicit or presumptive consent [5], in both cardiocirculatory death and brain death, requires extensive and repeated information of the general public about the resumption of non-heart-beating donation. Indeed, most people are probably not adequately informed and, consequently, cannot refuse or accept to be donors with full knowledge of what is involved. It is worth pointing out that implied consent does not refer specifically to brain death or cardiocirculatory death but to organ donation after death. The debate should probably take place at the level of the population, which, in a democracy, should be in charge of these fundamental issues that deal with the therapeutic relationship, the role for medicine, and the status of the individual as a human being. The introduction of Maastricht type III donation provides an opportunity for taking the debate beyond the healthcare community and for ensuring transparency of all transplantation activities in general. There is an opening for organizing an exhaustive information campaign with all the learned societies involved, the authorities responsible for regulating transplantation activities, organ retrieval and transplantation networks, patients on transplantation lists, healthcare professionals involved with transplantation, legislators, and the general public, to clarify issues raised by organ retrieval and the conduct of procedures for organ retrieval, including the legal, technical, and moral requirements.

Transparency at all the steps of the process would have to be ensured via sufficient media attention. A ruling from the national advisory ethics committee (CCNE) would be helpful.

## Methods

### Treatment withdrawal must comply with French law and with SRLF recommendations

ICU patients in whom death is deemed unavoidable should receive palliative care during the treatment-withdrawal process, in compliance with the Léonetti law and under the conditions described in SRLF recommendations [6]. These requirements ensure that the patient receives high-quality end-of-life management. The main points of the Léonetti law are as follows: unreasonable obstinacy is proscribed; the patient’s wishes should be taken into account, directly or indirectly; a collegial discussion must be held before the decision, which is made by the physician in charge of the patient; palliative care must be provided; and the entire procedure must be recorded (in a way that enables traceability) in the medical chart. The advice of an external consultant must be obtained to ensure the legitimacy of the procedure, in compliance with the law. The spirit of the law resides in avoiding both euthanasia and futility and in humanizing the dying process.

### When talking with families, information about treatment withdrawal should be clearly separated from information about organ retrieval

As stated in the introduction, this principle is crucial to ensure that the intensivist can make a treatment-withdrawal decision in a manner that is completely independent from potential Maastricht type III donation.

#### Supplying information about treatment withdrawal is among the intensivist’s normal duties

Information given to the family about the severity of the patient’s condition and about treatment-withdrawal decisions must be separated from the information about potential organ donation. The SRLF recommends following an information sequence similar to that suggested for stroke patients, as well as the very early delivery of information, at admission of the patient to the ICU: (a) the family is informed about the severity of the situation, and (b) explanations are given about the strong likelihood of treatment withdrawal depending on the patient’s clinical condition and under the usual conditions (collegial discussion, family conference, advice from a consultant, and detailed description of the process in the patient’s medical record).

Nevertheless, there are two situations in which the issue of separating the treatment-withdrawal decision from potential organ donation is not really relevant: (a) availability of a document (e.g., an organ donor card) in which the patient indicates his or her desire to be an organ donor (at present, this type of document seems applicable only in the event of brain death, but its relevance may be extended subsequently to Maastricht type III donation); (b) and spontaneous disclosure by the family, during or just after the family conference about treatment withdrawal, that the patient previously expressed a desire to be an organ donor. However, under no circumstances does this form of consent detract from the duty to first inform patients about all organ retrieval modalities, including Maastricht type III.

Here, the intensivist informs the family that treatment withdrawal is expected to be followed by the death of the patient. There is no reason to act differently in situations where organ donation may be considered than in the general situation described in the Léonetti law and SRLF recommendations, before the emergence of the Maastricht type III issue. As always, the procedure must be followed scrupulously; in particular, obtaining the advice of an external consultant contributes to the legitimacy of the treatment-withdrawal decision, which must be made independently from any considerations about potential organ donation.

#### Who should inform the family that organ donation is a possibility?

This point of the procedure is particularly sensitive. The knowledge that treatments will be withdrawn generates profound emotional distress in the family members. Indicating that organs may be collected is probably an aggravating factor (except when the family spontaneously considers this possibility or accepts immediately when the issue is raised). The respect due to the dying person and the duty to provide care to the family, together with the requirement that treatment-withdrawal decisions be made independently from organ retrieval decisions, provide both arguments for and arguments against each of the two available options: to have the issue of organ donation raised initially by the healthcare team or by the transplant coordinator team. In both situations, the healthcare team must be closely involved in the interviews and in the procedure.

Having the healthcare team raise the issue of organ donation ensures continuity of the relationship with the family and facilitates the intervention of the transplant coordinator team, which occurs immediately afterward, particularly when the family spontaneously brings up the possibility of organ donation. A potential disadvantage, however, is that the family may become unsure or even suspicious about the legitimacy of the treatment-withdrawal decision. On the other hand, in theory, having the issue raised by the transplant coordinator team in the presence of the healthcare team may underline the independence of the treatment-withdrawal decision from a desire to enable organ retrieval but also may raise questions in the minds of the family members.

The SRLF has not definitively stated that one or the other of these two possibilities is best. Both possibilities should be considered on a case-by-case basis. In every case, when the family is unprepared and has not requested organ donation, the possibility of Maastricht type III donation should be raised in a sensitive manner that will avoid a rejection response.

The timing of the investigations needed to determine whether organ donation is contraindicated is a difficult issue: should the investigations be done before the patient dies and before the family is informed or only after consent of the family is obtained? Is it logical and cost-effective to perform these investigations without knowing whether the families will consent to organ donation? In theory, there are two available options.

A reasonable advantage of performing the investigations (jointly with the transplant coordinator team) before informing the family is protection of the family from a trying phase of uncertainty. The results of liver and kidney ultrasonography and viral serologies indicate whether organs can be collected after the patient dies. This sequence has the theoretical advantage of not subjecting the family to the double ordeal of first discussing potential organ donation then learning that donation is not feasible. Testing for the HIV without consent from the family is a drawback, however; in addition to the absence of consent, which is required for this test, a positive result may generate distress of greater magnitude than that whose avoidance was intended. The physician must either inform the family of the positive HIV result, shown by a test that was performed without consent and unrelated to patient management (but that was done under the hypothesis of organ retrieval, which contradicts the principle that underlies our thinking) or fail to disclose the result, as if the patient had refused disclosure, except that the wishes of the patient in this regard are unknown.

Performing the investigations only after telling the family about the possibility of organ donation has the advantage, should consent to donation be given, of obtaining permission from the family to collect samples and implies that the results will be communicated to the family, including those of the HIV tests. These results may generate distress or disappointment, thereby jeopardizing the achievement of sufficient serenity to initiate the grieving process under favorable conditions. Nevertheless, given the potential disadvantages of the other option, full information of the family seems to comply best with both the legal and the ethical requirements that govern organ donation.

A ruling from the national advisory ethics committee (CCNE) would probably make a major contribution to this issue.

Depending on the option that was chosen, the healthcare team or the transplant coordinator team in the presence of the healthcare team will perform the following.

(i) Inform the family that organ donation is being considered to benefit patients who are on transplant waiting lists. If the patient expressed the wish to be a donor or the family spontaneously considered this possibility, instead of information that organ donation may occur, the family should receive information on donation modalities, as indicated below.

(ii) Before extubating the patient, obtain the explicit consent of the family to organ retrieval. Again, the modalities of this information should be adapted depending on the wishes expressed earlier by the patient or family.

The approach advocated by the SRLF is not different from the good practice rules to be followed when collecting organs from brain-dead patients, as outlined below.

Any wishes expressed explicitly by an adult patient (donor card, which can be likened to an advance directive for multiorgan retrieval; and verification of the donation refusal registry) should be honored. In this situation, collecting organs honors the patient’s right to autonomy. The relationship with the patient is not severed: instead, organ retrieval continues the therapeutic relationship by honoring the wishes the patient expressed as an autonomous individual. The technical procedure, far from inducing instrumentalization, serves the patient by respecting a choice he or she made before dying. In the setting of Maastricht type III donation, an important issue is whether consent given at a time the individual is healthy is applicable to the end of life. Wishes expressed previously by the patient are not sufficient, as the subsequent experience of a serious illness can profoundly modify the patient’s value system, leading to a change in the way he or she envisions the end of life. Far from resulting in a static construction, thinking evolves continuously in response to the life experiences it builds on.

If the patient did not express any wishes about organ donation before dying, and if the family did not raise the issue spontaneously, a family conference is in order. The rationale for obtaining informed consent from the family to organ retrieval and donation is that the family does not act based on their own wishes but instead based on what the dying patient would say if he or she were able to communicate. In this setting, every effort should be made to honor the patient’s wishes, if any were expressed clearly, which amounts to obtaining explicit consent.

(iii) Inform the family of the organ retrieval modalities and of the attendant requirements, particularly regarding the location where death will occur. The best location may be the operating room, for reasons related both to technical constraints and to the geographic distance between the ICU and the operating room. (see section below: where should the patient be extubated?)

(iv) Caution the family that the organ retrieval procedure may fail if the time from extubation to cardiocirculatory arrest is too long, which does not preclude the donation of corneas.

(v) Indicate to the family that the healthcare team is available for information about the necessary paperwork and that the family is free to withdraw their consent at any time.

### Treatment withdrawal modalities

#### Terminal extubation or terminal weaning?

The possibility of Maastricht type III donation should not lead to standardization of practices across ICUs, as the approach and sensitivity of each team are unique.

Although most teams perform extubation after a phase of preparation, the important point here is that the introduction of Maastricht type III donation should not affect the treatment-withdrawal procedures usually followed in each unit and, above all, that regardless of the method used, the tube is removed by the physician in charge of the patient.

#### Sedation

Sedation objectives and practices should be identical to those used in patients for whom organ donation is not being considered. If the situation requires sedation, SRLF recommendations should be followed: lethal injections are prohibited but medications known to exert a double effect can be used.

#### Where should the patient be extubated?

##### Extubation in the operating room

Advantage: the operating room is the best location for optimizing organ retrieval conditions.

Disadvantage: the high-technology and ultra-medicalized nature of the operating room has connotations of unfeelingness that are in complete contradiction with attempts to introduce a culture of palliative care in ICUs.

##### Extubation in the intensive care unit

Advantage: it is symbolically important to adhere strictly to usual practice and to deliver continuous care to the families.

Disadvantage: after death occurs, the patient’s body must be wheeled rapidly through the hallways to the operating room, creating an atmosphere of urgency that is not conducive to the serenity desirable under these circumstances. Extubation in the ICU may *per se* contribute to the failure of organ retrieval, thereby jeopardizing the reciprocal commitments made by the healthcare team to the family and by the family to the last wishes of the patient.

When extubation is performed in the operating room, the physician in charge of the patient accompanies the patient to the operating room and removes the tube. As with family information about possible organ donation, each situation should lead to an appropriate individual decision that strikes the best compromise between, on the one hand, respect of the patient and expectations of the family and, on the other, optimization of organ quality. When the patient expressed the wish to be an organ donor or the family spontaneously suggested organ donation, the focus is on respecting this altruistic attitude and, therefore, on optimizing the conditions of organ donation. On the opposite, exacerbated distress among family members and a desire to proceed exactly as usual when a patient dies after treatment withdrawal may prompt a decision to perform extubation in the ICU. Clearly, determining which decision is best requires close involvement of the family.

Thus, each center accredited by French organ transplantation authorities could be allowed to determine, in tight collaboration with the close relatives, which modalities should be given preference, depending on the relatives’ opinion and on favorable or unfavorable technical and architectural factors (distance from the ICU to the operating room). It is worth pointing out that some teams [7] always perform extubation in the ICU.

#### Providing continuous care to the family

Providing care to the family [8] is mandatory, not only to comply with the law, but also as part of the duty to alleviate the distress of people dealing with two devastating events:

a) loss of hope that the patient may survive, when treatment withdrawal is announced; and

b) information that organs may be collected and donated.

The place where the patient dies is important. Patient care—in the absence of organ retrieval—is usually provided in the ICU until death occurs, in the presence of the healthcare team (physicians and other healthcare professionals) and close relatives. The disadvantages of the operating room from a human standpoint have been described earlier. Nevertheless, organ retrieval results in constraints that have been described to, and accepted by, the family. This previous information step is crucial to remain consistent with the spirit of the Léonetti law, which seeks increased humanity and decreased medicalization of death.

### The intensivist certifies death by cardiocirculatory arrest

When the physician certifies death, he or she observes a state of the body, determines its cause, and predicts that this state is permanent. Although in the current state of the art, medicine can determine a probability that death is irreversible, it is the society in which the physician practices that equates this probability with a certainty.

The diagnosis of brain death rests on a detailed description of well-recognized clinical criteria and findings from investigations. In contrast, the diagnosis of death by cardiocirculatory arrest continues to raise major issues. Thus, laws vary across countries regarding the time from circulatory arrest to signature of the death certificate: for Maastricht type III patients, 2 min must be allowed to elapse in Pennsylvania, 5 min in Canada and France, 10 min in other states of the United States and the United Kingdom, and 20 min in Sweden.

The key point is the irreversible nature of death. The SRLF advocates adherence to the recommendations issued by the Institute of Medicine and Canadian Council for Donation and Transplantation [9]: to certify that irreversible death has occurred, the physician must wait 5 min after the start of cardiocirculatory arrest, without any attempts at resuscitation, to confirm beyond all doubt that spontaneous resumption of cardiac function capable of restoring circulation will not occur. Auto-resuscitation has never been reported to occur after more than 65 s of circulatory arrest: after this time (and even more so after 5 min), the cessation of circulatory and respiratory function is permanent (“will not return”) and then inevitably and rapidly becomes irreversible (“cannot return”).

### Organ retrieval rules for Maastricht type III donation

#### The dead donor rule

Adhesion to the dead donor rule [10] implies that the dying patient (who is still alive) is not instrumentalized: cardiopulmonary bypass cannot be started as long as the patient is alive. The SRLF strongly believes that cannulation (if required) cannot be considered until death is certified, which removes all possibility of transgression at this stage of the procedure. This restriction may decrease the number of transplants. Organ retrieval from a patient who has died within this ethical and regulatory framework is neither a profanation nor a transgression, as the technical procedures performed on the dead body are designed only to preserve the organs intended for transplantation.

The ability to predict the time to death by circulatory arrest after the withdrawal of life-sustaining treatments is crucial. Indeed, this time determines the risk of organ deterioration, on which depends the feasibility of collecting organs for transplantation. This concern about transplant quality before the death of the patient clearly reflects the ambivalence of the medical viewpoint and the risk of treating the patient as an object before death occurs, as discussed above.

Organ retrieval requires a duration of warm ischemia (systolic blood pressure < 50 mmHg) no greater than 90 min, or perhaps even 60 min. The temptation may arise to shorten the warm ischemia time with the goal of preserving organ function should cardiac arrest fail to occur promptly after extubation. To achieve this goal, instead of allowing death to occur naturally, actions would have to be taken to hasten death, which would very probably affect the grieving process in the family members. Therefore, the SRLF considers that hastening death is unethical, because palliative care would then no longer be the priority, and should not be performed.

#### Destination of the body after organ retrieval

After organ retrieval, the destination of the body is, in principle, the same as after other circumstances of organ collection (for instance after brain death).

#### Provisional accreditation for organ retrieval in pioneer centers initially

It seems necessary that accreditation for Maastricht type III organ retrieval be provisional initially. Accreditation could occur as a temporary authorization procedure, as was the case for Maastricht types I and II organ retrieval.

#### Need for an evaluation by French transplantation authorities (agence de la Biomédecine, ABM)

The SRLF advocates having the transplant coordinator be the guarantor of application of good practice guidelines, in keeping with some of the proposals by the ABM. However, a legitimate concern is whether this approach might have a risk of conflicts of interest. A more relevant course of action might be to obtain an independent evaluation by the ABM of both transplantation procedures and the indications of organ donation.

### Management of critical situations

#### Failure of organ retrieval

Failure of organ retrieval occurs when the time from extubation to circulatory arrest is too long to allow the collection of viable organs. That this situation may arise should be discussed with the family before the procedure, as indicated above.

#### Conflict of interest between the family and the healthcare team

There is a risk that the family might suspect the healthcare team of deciding to withdraw life-sustaining treatments with the goal of enabling organ retrieval. Therefore, as pointed out above, the procedure for treatment withdrawal must be followed scrupulously.

#### Moral difficulties generated by the introduction of Maastricht type III donation

Although latent concern among family members about possible instrumentalization can never be completely eliminated, the healthcare team must be absolutely convinced that instrumentalization does not occur. To this end, the healthcare team must continuously monitor the ethical appropriateness of its actions by answering the following questions.

1) Was the attention given to the vital interests of the potential donor free from any considerations about possible organ retrieval?

2) Was care to the donor viewed as the finality of care, as opposed to a means, at the initial phase of his or her management?

3) Has the possibility of care and procedures being performed to improve the organ supply been convincingly eliminated?

4) Was there, at any time, a “strategic” use of futile care aimed at improving the likelihood of successful organ retrieval?

Scrupulously applying the intangible principles set forth above is paramount in a setting where the ICU team may feel that the final goal has changed: instead of acting only for the patient, actions directed toward another target may be taken, not *subsequently*, but *at the same time*. Avoiding such confusion is among the main goals of the principles set forth in this document.

#### The vulnerable patient

Patients who seem to have no family or close friends raise difficult problems. In this situation, applying the implicit consent principle advocated by French law but rejected in the present document raises obvious moral concerns. Involvement of a third party (e.g., a guardianship judge, as for the protection of vulnerable adults) might deserve consideration. Here also, a ruling by the national advisory ethics committee (CCNE) would be helpful.

#### Conflicts of duty

Two major sources of ethical tension may arise in units authorized to manage potential Maastricht type III donors. One may result from a feeling of frustration due to the low organ retrieval rate when recommendations similar to those put forth in this document are applied. In this case, a utilitarian perspective may seem to hold appeal as a means of increasing the rate of successful organ retrieval, which may lead to premature withdrawal of active treatments or, on the contrary, to the “strategic” use of futile treatments until the technical conditions for organ retrieval are met. The other source of tension is related to the possible perception among some of the physicians or other healthcare professionals that, despite all the precautions described above, they contributed to what they perceive as a transgression. Clearly, these potential tensions will have to be monitored and evaluated. Thus, ICUs participating in the pilot phase of Maastricht III donation should be audited routinely, not only to evaluate the procedures but also to analyze the experience of all those involved in the process, namely, the ICU team (physicians and nurses), the donor transplant coordinator team, and the families of Maastricht type III donors. These audits will have to be conducted by an independent agency to ensure that the responses of the interviewees are not biased.

This evaluation phase will be crucial to ensure that the procedure is conducted properly, to adjust practices, to review recommendations, and to improve the experience of all those involved.

## Endnotes

### ^*^For the ethics committee of the SRLF

Didier Dreyfuss (secretary), Pierre-Edouard Bollaert, Laurence Bloch, Ludivine Chalumeau-Lemoine, Robin Cremer, Cédric Daubin, Dominique Folscheid, Jean-Pierre Graftieaux, Olivier Guisset, Lise Haddad, Philippe Hubert, Didier Journois, Nancy Kentish-Barnes, Alexandre Lautrette, Guy Le Gall, Anne Renault, Christian Richard, René Robert, Marina Thirion, and Daniel Villers.

## Abbreviations

ABM: Agence de la biomédecine; CCNE: Comité consultatif national d’éthique; DCDD: Donation after circulatory determination of death; HIV: Human immunodeficiency virus; ICU: intensive care unit; SFMU: Société française de médecine d’urgence; SFAR: Société française d’anesthésie et de réanimation; SRLF: Société de réanimation de langue française.

## Competing interests

The authors declare that they have no competing interests

## Authors’ contributions

JPG drafed the manuscript. PEB, LH, NKB, GN, RR, DV and DD critically revised the manuscript for important intellectual content. All authors read and approved the final manuscript.
